# Supplementary material for: Cannabidiol blood metabolite levels after cannabidiol treatment are associated with broadband EEG changes and improvements in visuomotor and non-verbal cognitive abilities in boys with autism requiring higher levels of support
Source: Transl Psychiatry. 2026 Jan 30;16:109. doi: 10.1038/s41398-026-03815-y (PMC12923786; doi:10.1038/s41398-026-03815-y)
Supplement: Supplementary file 1 — Supplementary Figure Legends [file 41398_2026_3815_MOESM1_ESM.docx]

**Figure Supplement 1. CBD blood metabolite levels and preprocessing of task-free EEG signals. (A)** Concentration of metabolite levels in blood, including CBD, **(B)** 7-OH-CBD and **(C)** 7-COOH-CBD, across all study timepoints. **(D)** Representative traces of segmented EEG before and after artifact rejection. **(E)** Representative aperiodic exponent and offset values for all EEG electrode channels in a single session for a single participant.

**Figure Supplement 2. Spectral parameterization frequency range comparison.** (A) Error distribution comparison across all channels shows that 0.5-13 Hz fits (teal) produce systematically lower model errors compared to 0.5-50 Hz fits (orange), with means of ~0.04 and ~0.06 respectively. (B) Box plot comparison showing the consistent reduction in model error with 0.5-13 Hz frequency restriction. (C) Representative examples demonstrate how 0.5-50 Hz fits (right column) attempt to model high-frequency artifacts, resulting in high frequency noise-biased aperiodic parameters compared to the 0.5-13 Hz fits (left column).

**Figure Supplement 3. Absent relationships between cognitive-behavioral assessments and CBD blood metabolite levels.** Results from linear mixed models and linear regressions relating (**A)** repetitive behavior, **(B)** expressive vocabulary assessment scores to levels of CBD, 7-OH-CBD, and 7-COOH-CBD metabolite in blood. Visualization of each linear mixed model shows the z-statistic for each coefficient and a heatmap of the two-tailed p-value associated with the z-statistic. #p<0.06.
