# Supplementary material for: Cannabidiol blood metabolite levels after cannabidiol treatment are associated with broadband EEG changes and improvements in visuomotor and non-verbal cognitive abilities in boys with autism requiring higher levels of support
Source: Transl Psychiatry. 2026 Jan 30;16:109. doi: 10.1038/s41398-026-03815-y (PMC12923786; doi:10.1038/s41398-026-03815-y)
Supplement: Supplementary file 2 — Figure Supplement 1 [file 41398_2026_3815_MOESM2_ESM.pdf]

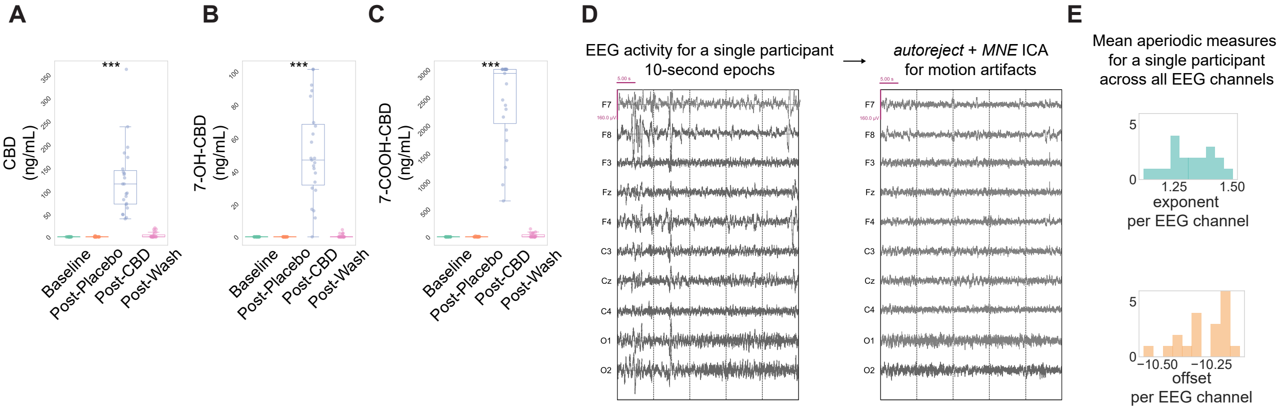
A
B
C
D
E
CBD (ng/mL)
7-OH-CBD (ng/mL)
7-COOH-CBD (ng/mL)
***
Baseline
Post-Placebo
Post-CBD
Post-Wash
EEG activity for a single participant
10-second epochs
autoreject + MNE ICA
for motion artifacts
F7
F8
F3
Fz
F4
C3
Cz
C4
O1
O2
Mean aperiodic measures
for a single participant
across all EEG channels
exponent
per EEG channel
offset
per EEG channel
1.25
1.50
−10.50
−10.25
