# Supplementary figures and images for: Cannabidiol blood metabolite levels after cannabidiol treatment are associated with broadband EEG changes and improvements in visuomotor and non-verbal cognitive abilities in boys with autism requiring higher levels of support
Source: Transl Psychiatry. 2026 Jan 30;16:109. doi: 10.1038/s41398-026-03815-y (PMC12923786; doi:10.1038/s41398-026-03815-y)

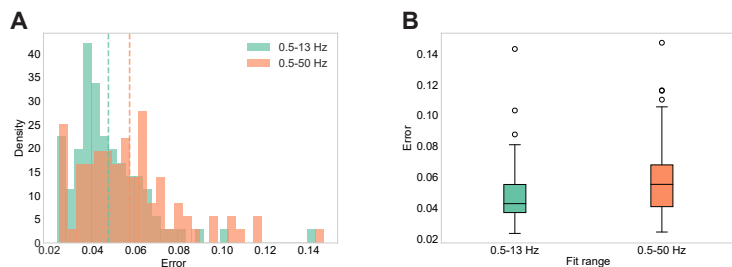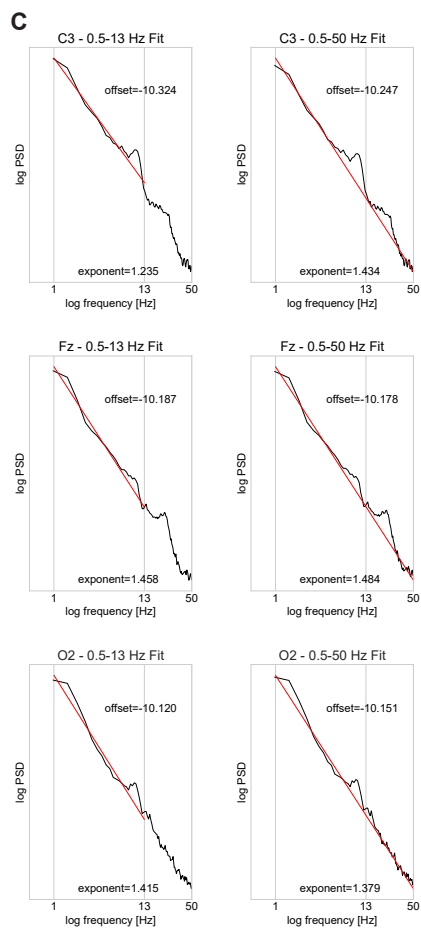

Supplement: Supplementary file 3 — Figure Supplement 2 [file 41398_2026_3815_MOESM3_ESM.pdf]

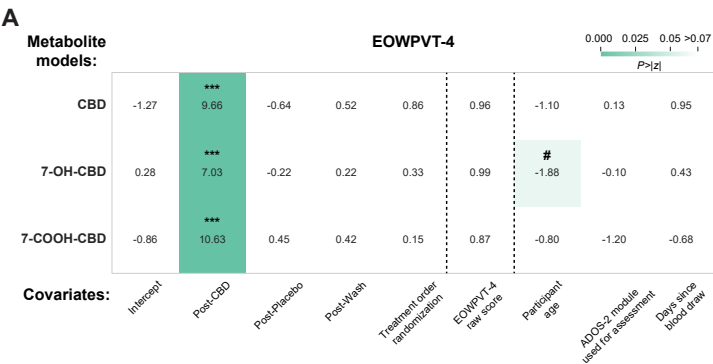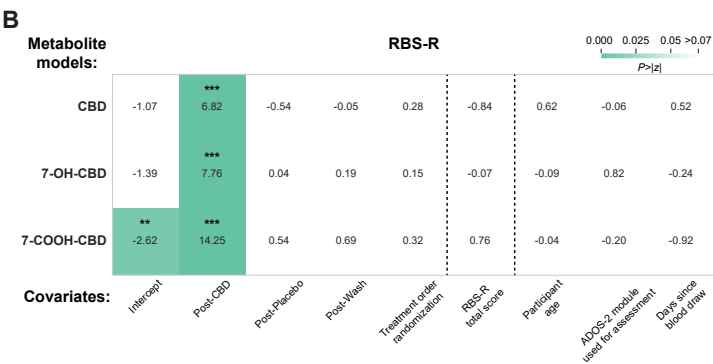

Supplement: Supplementary file 4 — Figure Supplement 3 [file 41398_2026_3815_MOESM4_ESM.pdf]
