# Supplementary material for: Cannabidiol blood metabolite levels after cannabidiol treatment are associated with broadband EEG changes and improvements in visuomotor and non-verbal cognitive abilities in boys with autism requiring higher levels of support
Source: Transl Psychiatry. 2026 Jan 30;16:109. doi: 10.1038/s41398-026-03815-y (PMC12923786; doi:10.1038/s41398-026-03815-y)
Supplement: Supplementary file 5 — Supplementary Materials [file 41398_2026_3815_MOESM5_ESM.docx]

**Patients and Methods**

**Experimental recordings and cognitive-behavioral testing**

**Participants**

Our data was sourced from a clinical trial performed at the University of California. The trial was approved by the institutional review board of the University of California, San Diego and registered with www.clinicaltrials.gov (NCT04517799) to investigate the effect of daily oral CBD (Epidiolex®) on problem behaviors in boys with severe symptoms of autism. Due to the subjects’ substantially limited cognitive capabilities, a waiver of child assent was approved for the study from which our data was sourced. Parental or guardian permission was obtained for each child’s enrollment and participation prior to their inclusion in the study from which our data was sourced. The parent or guardian also provided consent for completing the questionnaires required for the study from which our data was sourced. The dataset included in this study consisted of 24 males (ages 7-14) who had prior clinical diagnoses of autism spectrum disorder (ASD) using DSM-5 criteria. The diagnoses were made by clinicians with expertise and experience in diagnosing and treating children with ASD. All participants underwent Autism Diagnostic Observation Schedule, Second Edition (ADOS-2) assessments of symptoms and exhibited severe behavioral issues including stereotypies, aggression, self-injury, and/or hyperactivity, as determined by a clinician. Participants had no current or historical seizure disorders or other neurological conditions. Participants were allowed to be on psychoactive medications (risperidone, etc.) if they had been on a stable dose for at least 3 months prior to randomization. Only 2 participants took the following after the initial assessment date (Baseline): one participant took magnesium glycinate and Dimetapp (non-drowsy) Oral Product, while the other took a M.V.I. Pediatric (multivitamin) and Benadryl. These participants were not excluded due to the minimal effect these medications would have on the measures collected in this study.

**Experimental design**

Our dataset consisted of assessments made via a randomized, two-arm design (CBD-Placebo or Placebo-CBD) with treatment periods lasting 8 weeks, separated by a 4-week washout period. Cognitive-behavioral tests and EEG recordings were conducted at four time points: Baseline, Post-placebo, Post-CBD, and Post-wash. The trial used Epidiolex®, an FDA-approved oral purified CBD solution developed by GW Research Ltd. for treating certain rare pediatric epilepsies. The dosage was adjusted based on participant weight, with a gradual increase over the first two weeks to reach the maximum dose (20 mg/kg/day, divided into two oral doses) for the remainder of the study period. Our analyses focused solely on the EEG data and cognitive-behavioral assessments collected during this trial, aiming to investigate the relationship between these measures and CBD metabolite blood levels.

CBD is metabolized to form the major active metabolite 7-hydroxy-cannabidiol (7-OH-CBD) and non-active metabolite 7-carboxy-cannabidiol (7-COOH-CBD). The dataset used in this study also included quantifications of whole blood samples near EEG and cognitive-behavioral assessments. For Post-CBD study time point, quantifications were taken an average of 2-3 days prior to assessments. The lower limits of quantification (LLOQ) in whole blood were: 0.5 ng/mL for CBD, for 7-OH-CBD, and for 7-COOH-CBD. The upper limits of quantification (ULOQ) in whole blood were: 500 ng/mL for CBD, 500 ng/mL for 7-OH-CBD, and 3000 ng/mL for 7-COOH-CBD. Values below the LLOQ of metabolites were implemented as 0 in our analyses, while those exceeding the ULOQ were analyzed as the max value of each metabolite. While this approach may have slightly underestimated very low concentrations and capped the highest concentrations, it provided us with a standardized way to deal with values outside the quantifiable range.

**Cognitive-behavioral testing**

Our dataset included cognitive and behavioral assessments from each time point: (1) Test of Nonverbal Intelligence, Fourth Edition (TONI-4), a language-free test that assesses intelligence in individuals with limited language abilities, (2) Repetitive Behavior Scale - Revised (RBS-R), a rating scale administered to the participant’s primary caregiver to report the frequency and severity of the repetitive and restricted behaviors that are a common feature in individuals with ASD, (3) Peabody Picture Vocabulary Test, Fourth Edition (PPVT-4), a measure of receptive vocabulary for Standard American English, (4) Expressive One Word Picture Vocabulary Test, Fourth Edition (EOWPVT-4), an assessment of the participant’s ability to name a variety of objects, actions, and concepts, (5) Beery-Buktenica Test of Visual Motor Integration, Sixth Edition (Beery VMI-6), a composite assessment of the participant’s ability to integrate their visual and motor abilities and includes supplemental subtests for visual perception (Beery VP) and motor coordination (Beery MC). We also used the ADOS-2 module (ranging from module 1-3) used to assess participants.

Standard scores were calculated where possible for sample characterization purposes (Table 1), while raw scores were used for longitudinal analyses of the EOWPVT-4, PPVT-4, and Beery VMI-6 assessments due to floor effects in our severely impaired sample. Floor effects limit the capacity of standard scores to reflect ability that falls below the lowest standard score, whereas raw scores provide a measure reflecting the fuller range of participant abilities. As demonstrated in Table 1, substantial proportions of participants who achieved standard scores performed at or near the test floor, and for these participants, any potential improvement would be undetectable using standard scores since they cannot fall below the lowest possible standardized value. Raw scores, however, can capture incremental gains above baseline performance, providing sensitivity to meaningful changes that would be missed by standard scores in treatment research with severely impaired populations.

**EEG recording setup**

Our dataset included recordings made using a 20-channel dry EEG wireless headset device configured in the 10-20 montage (CGX, formerly Cognionics, Quick-20 Dry EEG Headset, https://www.cgxsystems.com). Electrodes were referenced online to the left ear clip (A1), and the right ear clip (A2) was recorded as an active electrode. EEG data were digitized with 24 bits of resolution at 500 Hz with a bandwidth of 0-131 Hz with true DC coupling. Impedance measurements were conducted at the start of each recording session using the CGX Quick-20 Dry EEG Headset’s built-in impedance monitoring system. The 500 kΩ threshold was selected based on the manufacturer’s specifications for their dry electrode technology. While this threshold is higher than traditional wet electrode systems (which typically target <50 kΩ), it was appropriate for this system due to the dry electrode design, which inherently has higher impedance than wet electrodes. EEG recordings were made during awake, passive viewing periods with the goal of capturing 5 to 10 minutes during which the child remained calm by viewing a youtube video of their choice.

**Data analysis**

Data analysis was performed with python using numpy [1.26.3], scipy [1.11.4], autoreject [0.4.3], MNE [1.6.0], and specparam (formerly known as FOOOF) [1.1.0] for EEG, and statsmodel for calculating linear mixed effects models [0.14.1]. A total of 19 EEG channels (F7, Fp1, Fp2, F8, F3, Fz, F4, C3, Cz, P8, P7, Pz, P4, T3, P3, O1, O2, C4, T4) were included in the analysis. Channels were also subdivided into frontal (F7, F3, Fz, F4, F8), central (Cz, C3, C4) and occipital (O1, O2) groups for post-hoc testing. The analysis code needed to reproduce the analysis and figures is provided here: https://github.com/voytekresearch/cbd-asd-eeg

**Preprocessing**

First, EEG data was digitally re-referenced offline to a linked ears reference composed of both A1 and A2 for analysis. We then implemented an automated cross-validation procedure via the Autoreject python package to estimate a within-session, “global” peak-to-peak voltage rejection threshold shared across all channels. This rejection threshold was then used to remove channels contaminated by excessive noise artifacts. The surviving channel data was then bandpass filtered between 0.1-100 Hz before use of the ICLabel independent component classifier integrated with MNE. In brief, to detect and remove ocular artifacts and eyeblinks, independent component analysis (ICA) was applied to the filtered data using the “extended infomax” method and to decompose the signal into the smallest number of components required to explain the 99% of the cumulative variance of the data. Components labeled as “brain” or “other” (i.e., non-classifiable components) were kept while all others, including labels that indicated ocular artifacts, were removed. Due to the small number of channels, ICA could not always remove ocular and muscular artifacts. Therefore, we epoched EEG data into 10-second segments and calculated “local” peak-to-peak rejection thresholds within these epochs using the same Autoreject python package procedures described above. This “local” approach estimates a rejection threshold for each channel within epochs, and depending on the number of highly contaminated channels, this epoch is repaired by interpolation or excluded from subsequent analysis. Surviving epochs then had a hamming window applied to account for epoch edge artifacts and discontinuity before undergoing concatenation. The preprocessing procedures used in this study were devised to account for the difficulty in maintaining a high quality of EEG data in ASD children who are prone to movement.

Exclusion criteria were: (1) Sessions with post-processed EEG data of < 30 seconds in duration (2) in cases where multiple EEG recording attempts were made due to disruption from the participant within a study time point, only the session with the highest average alpha power signal-to-noise-ratio estimated from channels O1 and O2 was used for further analysis. This approach was necessary for 6 of our 24 participants, two of whom required up to 4 attempts at a single timepoint to obtain EEG without removing their headsets. In the end, a total of 89 EEG testing sessions (Baseline: 21, Post-CBD: 23, Post-Placebo: 23, Post-Wash: 22) were included in this study, with 3-4 recordings per child corresponding to each of the 4 study time points.

**Calculation of EEG signal features**

The power spectral density (PSD) was estimated from EEG signals using Welch’s method using 1.0 s Hamming windows with 0.5 s overlap within the frequency range 0.1-50 Hz. High levels of muscle noise are indicated by elevated power levels above 10 Hz in the spectral domain. To minimize the contamination of the exponent model fit due to muscle noise, we parameterized the spectra within the 0.5-13 Hz frequency range. Thus, the settings for the spectral parameterization algorithm were: peak width limits: (0.5, 13.0); maximum number of peaks: 6; minimum peak amplitude exceeding the aperiodic fit: 0.0; peak threshold: 2.0; and aperiodic mode: ‘fixed’. This resulted in estimates of the aperiodic exponent, aperiodic offset, and aperiodic adjusted delta (0.1-4 Hz), theta (4-8 Hz), and alpha (8-13 Hz) power. Only channels with model fits satisfying a minimum R^2^ value of 0.80 were kept for further analysis. To quantify the strength of neural oscillations, we calculated the Signal-to-Noise Ratio (SNR) for each frequency band of interest (Delta, Theta, and Alpha). In brief, for each channel and frequency band, we computed an aperiodic-corrected PSD by subtracting the modeled aperiodic fit from the original PSD. Within each frequency band, we identified the maximum value of the corrected PSD, which we defined as the SNR for that channel and band. To obtain a global measure, we calculated the mean SNR across all valid channels for each frequency band. This approach allowed us to quantify the strength of oscillations in each frequency band relative to the aperiodic background, providing a robust measure of band-specific neural activity across the scalp. The resulting SNR values are expressed in units of power spectral density (µV²/Hz) and reported as aperiodic adjusted power, representing the difference between the peak oscillatory power and the aperiodic background power in each frequency band.

**Statistical analysis**

We z-scored metabolite blood levels across subjects to account for non-normality caused by floor and ceiling effects from LLOQ and ULOQ detection thresholds. For statistical evaluation, Linear Mixed Effects (LME) models were built to investigate the association between z-scored CBD metabolite levels in blood samples and (1) EEG signal features or (2) cognitive-behavioral testing outcomes. Our decision to model CBD metabolite levels as dependent variables was driven by multicollinearity considerations. CBD metabolites are highly intercorrelated due to sequential metabolism (CBD to 7-OH-CBD to 7-COOH-CBD), and EEG measures are similarly correlated within electrode groups as they derive from the same neural signals. Including multiple correlated metabolites or EEG features as simultaneous coefficients would violate linear regression assumptions due to multicollinearity, leading to inflated standard errors and unstable coefficient estimates. For cognitive-behavioral assessments, most measures served as single coefficients, but we maintained the same analytical framework (metabolites as outcomes) to ensure consistency across all analyses and enable direct comparison of effect sizes and significance patterns between EEG and cognitive domains.

Linear mixed-effects models were fitted using the smf.mixedlm function from the Statsmodels library [0.14.1], with a subject grouping variable as a random intercept term to account for within-subject correlations across study time-points. To account for variance explained by individual differences and treatment conditions, fixed terms included the randomization order (CBD first or placebo first) and the time point of EEG and cognitive-behavioral assessments (Baseline, Post-placebo, Post-CBD, Post-wash). Additionally, to account for subject characteristics that might affect CBD metabolism or cognitive-behavioral assessments, we included fixed terms for age, ADOS-2 module used for assessment, and amount of days from blood draw to assessments.

To test the association between CBD metabolite levels in blood samples and EEG signal features, we included fixed terms for aperiodic offset, aperiodic exponent, and aperiodic-adjusted signal-to-noise ratios in the delta, theta, and alpha bands. Thus, the LME model for CBD metabolite levels in blood against EEG signal features consisted of the following formula:

Mz = β0 + β_T_(T) + β_Rand_(Rand) + β_Off_(Off) + β_Exp_(Exp) + β_Delta_(Delta) + β_Theta_(Theta) + β_Alpha_(Alpha) + β_Age_(Age) + β_ADOS_(ADOS) + β_TIME_(TIME) + (1|SUBJ) + εi

Where *Mz* is the z-score of the CBD metabolites, *T* the study time point (Baseline, Post-placebo, Post-CBD or Post-wash), *Rand* is the randomization condition (CBD first or placebo first), *Off* is the aperiodic offset, *Exp* is the aperiodic exponent, *Delta* is the aperiodic-adjusted delta SNR, *Theta* is the aperiodic-adjusted theta SNR, *Alpha* is the aperiodic-adjusted alpha SNR, *Age* is the age of the participant, *ADOS* is the subject’s corresponding ADOS-2 module used for assessment, and *TIME* is the amount of days from blood draw to assessments. *βx* is the linear regression coefficient for each corresponding fixed effect term (β0 being the intercept term). *(1|SUBJ)* represents the random intercept term for each participant, allowing for individual variation in the overall blood level of the metabolite. εi is the error term. This model was applied separately to each CBD metabolite and repeated for each electrode group (Frontal, Central and Occipital). This approach allowed us to investigate whether the relationships between EEG signal features and CBD metabolite levels differed across the scalp.

To investigate the association between metabolite levels in blood samples and cognitive-behavioral assessments, we adapted our model to include fixed terms corresponding to the score of each assessment. Thus, the LME model for CBD metabolite levels in blood against the cognitive-behavioral assessment scores consisted of the following formula:

Mz = β0 + β_T_(T) + β_Rand_(Rand) + β_Score_(Score) + β_Age_(Age) + β_ADOS_(ADOS) + β_TIME_(TIME) + (1|SUBJ) + εi

Where *Mz* is the z-score of the CBD metabolite, *T* is the study time point (Baseline, Post-placebo, Post-CBD or Post-wash), *Rand* is the randomization condition (CBD first or placebo first), *Score* is the assessment score, *Age* is the age of the participant, *ADOS* is the subject’s corresponding ADOS-2 module used for assessment, and *TIME* is the amount of days from blood draw to assessments. *βx* is the linear regression coefficient for each corresponding fixed effect term (β0 being the intercept term). *(1|SUBJ)* represents the random intercept term for each participant, allowing for individual variation in the overall blood level of the metabolite. εi is the error term. This model was applied separately for each assessment (TONI-4, RBS-R, PPVT-4, EOWPVT-4, Beery VMI-6, Beery VP, and Beery MC). This approach allowed us to investigate whether the relationships between cognitive-behavioral assessment scores and CBD metabolite levels differed across various domains of cognition and behavior being assessed.

All model fitting was performed using the Nelder-Mead optimization method, with a maximum of 1000 iterations to ensure convergence. A Variance Inflation Factor (VIF) was calculated for each explanatory variable to assess multicollinearity and determine whether correlated variables should be combined or removed from the model (e.g. variables above a VIF of 10). We extracted coefficients, standard errors, z-values, z-statistic associated p-values, and 95% confidence intervals from model outputs to determine post-hoc linear regression analyses on each significant coefficient’s estimated marginal means in the Post-CBD time point. The number of sessions included in each model are listed in Table 1.

When significant associations were detected in the mixed models, we conducted post-hoc linear regression analyses using estimated marginal means to visualize the directionality and magnitude of these relationships. These post-hoc analyses examined simple bivariate relationships without controlling for covariates, serving as visualization aids.
